# Supplementary material for: Splice-Junction-Based Mapping of Alternative Isoforms in the Human Proteome
Source: Cell Rep. Author manuscript; Available in PMC 2020 Jan 15. (PMC6961840; doi:10.1016/j.celrep.2019.11.026)
Supplement: 2 [file NIHMS1546469-supplement-2.zip › DF1/PXD009737/TestisTrypsin/TestisTrypsin_62_GPS1_VGWLEGGQAHGVWGLVAVSCSLSPLQSPPR.pdf]

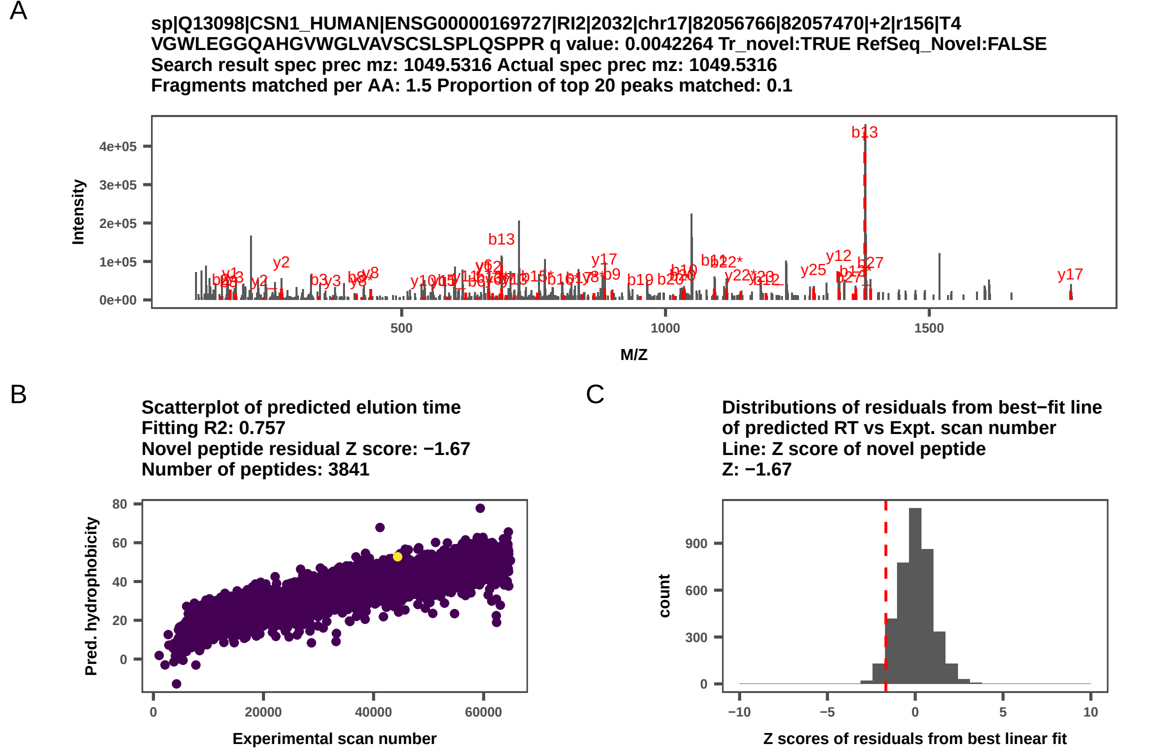
A
sp|Q13098|CSN1_HUMAN|ENSG00000169727|RI2|2032|chr17|82056766|82057470|+2|r156|T4
VGWLEGGQAHGVWGLVAVSCSLSPLQSPPR q value: 0.0042264 Tr_novel:TRUE RefSeq_Novel:FALSE
Search result spec prec mz: 1049.5316 Actual spec prec mz: 1049.5316
Fragments matched per AA: 1.5 Proportion of top 20 peaks matched: 0.1
Intensity
4e+05
3e+05
2e+05
1e+05
0e+00
500
1000
1500
M/Z
B
Scatterplot of predicted elution time
Fitting R2: 0.757
Novel peptide residual Z score: −1.67
Number of peptides: 3841
Pred. hydrophobicity
80
60
40
20
0
0
20000
40000
60000
Experimental scan number
C
Distributions of residuals from best−fit line
of predicted RT vs Expt. scan number
Line: Z score of novel peptide
Z: −1.67
count
900
600
300
0
−10
−5
0
5
10
Z scores of residuals from best linear fit
